# Calcium connections: endoplasmic reticulum Ca^2+^ homeostasis drives spontaneous Ca^2+^ oscillations in guard cells

**DOI:** 10.1111/nph.70961

**Published:** 2026-01-30

**Authors:** Jon K. Pittman

**Affiliations:** ^1^ School of Natural Sciences, Faculty of Science and Engineering The University of Manchester Manchester UK

**Keywords:** blue light photoreception, Ca^2+^‐ATPase, calcium, calcium imaging, calcium oscillations, calcium signalling, endoplasmic reticulum, guard cells

## Abstract

This article is a Commentary on Grenzi *et al*. (2026), **250**: 2852–2866.

Elevations and oscillations in concentrations of cytosolic calcium ions ([Ca^2+^]_cyt_) are known to occur in nearly all cell types across life (Edel *et al*., 2017). In many cases, these [Ca^2+^]_cyt_ elevations are induced by specific environmental or developmental stimuli that can give rise to precise spatio‐temporal patterns of [Ca^2+^]_cyt_ elevation (or Ca^2+^ signatures) that act as signals to transduce an appropriate downstream response (McAinsh & Pittman, 2009; Tian *et al*., 2020). In addition to stimulus‐induced [Ca^2+^]_cyt_ elevations, so‐called ‘spontaneous’ [Ca^2+^]_cyt_ elevations have been observed, particularly in model systems, such as stomatal guard cells (Siegel *et al*., 2009), with repetitive [Ca^2+^]_cyt_ oscillations being a marker of stomatal aperture closure (Minguet‐Parramona *et al*., 2016). While substantial pioneering research studies have identified many of the components that can encode and decode Ca^2+^ signatures, including Ca^2+^ influx and efflux transporters, buffers and signalling molecules (Edel *et al*., 2017; Tian *et al*., 2020; Brownlee & Wheeler, 2025), gaps in our understanding remain. This is especially the case for guard cell spontaneous [Ca^2+^]_cyt_ oscillations, where the mechanisms for their generation and maintenance have remained unclear. In an article published in this issue of *New Phytologist*, Grenzi *et al*. (2026; pp. 2852–2866) convincingly demonstrate that spontaneous [Ca^2+^]_cyt_ oscillations in *Arabidopsis thaliana* (Arabidopsis) guard cells are generated using the extracellular apoplastic Ca^2+^ pool, while the maintenance of these oscillations over time is dependent on the dynamics of Ca^2+^ release and refilling at the endoplasmic reticulum (ER).

*This study exemplifies the benefits of simultaneous subcellular Ca* ^*2+*^ *imaging in multiple locations to further enhance our understanding of Ca* ^*2+*^ *oscillations and Ca* ^*2+*^ *signalling mechanisms*.

Cellular Ca^2+^ homeostasis involves interacting processes across the whole cell. The generation of [Ca^2+^]_cyt_ oscillations often requires a source of Ca^2+^ from the apoplast as well as Ca^2+^ uptake and release from intracellular stores, notably the ER and vacuole (Brownlee & Wheeler, 2025). Ca^2+^ transfer into other organelles can also be important, and Ca^2+^ transients and oscillations have been observed in the ER, mitochondria, Golgi apparatus, chloroplast and nucleus (Resentini *et al*., 2021b). Previous studies had confirmed that apoplastic Ca^2+^ was required for generating spontaneous guard cell [Ca^2+^]_cyt_ oscillations (Siegel *et al*., 2009), but the importance of intracellular Ca^2+^ stores was unclear. The combined imaging of Ca^2+^ in multiple locations can be used to determine the involvement of intracellular compartments, such as the ER in Ca^2+^ homeostasis and signalling (Resentini *et al*., 2021a; Guo *et al*., 2022). Grenzi *et al*. therefore made use of Arabidopsis lines expressing two genetically encoded fluorescent Ca^2+^ indicators to simultaneously quantify [Ca^2+^]_cyt_ and ER lumen Ca^2+^ ([Ca^2+^]_ER_) dynamics in the same guard cell. To achieve high sensitivity of Ca^2+^ imaging and differential excitation in both subcellular locations, a cytosolic localised, red‐shifted R‐GECO1, which is excited by green light and an ER localised, green‐shifted ER‐GCaMP6‐210 excited by blue light, were used. This allowed the detection of spontaneous [Ca^2+^]_ER_ oscillations alongside the spontaneous [Ca^2+^]_cyt_ oscillations. However, the interplay between the two compartments was complex: in some cases, there was a delayed increase in [Ca^2+^]_ER_ following a [Ca^2+^]_cyt_ transient; sometimes, the Ca^2+^ increases in both the cytosol and ER were simultaneous; and in other cases, [Ca^2+^]_ER_ decreased following a [Ca^2+^]_cyt_ increase (Grenzi *et al*.).

Further analysis was required to determine whether the observed [Ca^2+^]_ER_ changes were simply a by‐product of the spontaneous [Ca^2+^]_cyt_ oscillations or an essential component. Despite the complexity of the [Ca^2+^]_ER_ patterns, on average, it was noticeable that the ER Ca^2+^ indicator fluorescence decreased over time, suggesting ER Ca^2+^ depletion, which typically coincided with an increase in cytosolic Ca^2+^. This is also consistent with a Ca^2+^‐induced Ca^2+^ release model of [Ca^2+^]_cyt_ oscillation generation (Xiong *et al*., 2025). However, a challenge with the use of the ER‐GCaMP6‐210 Ca^2+^ reporter is that it requires blue light excitation, yet blue light perception, especially through the action of phototropin (PHOT1 and PHOT2) blue light receptors, is known to control an array of processes in guard cells, including pathways thought to trigger Ca^2+^ release from internal stores (Kostaki *et al*., 2020). Grenzi *et al*. therefore repeated imaging experiments under different light and dark conditions. It was confirmed that blue light induces the release of Ca^2+^ from the ER, while a period of darkness allows refilling of the ER Ca^2+^ pool. Use of the *phot1*/*phot2* mutant line indicated that PHOT1 and/or PHOT2 are partly responsible for ER Ca^2+^ depletion, and seemingly in a kinase‐dependent manner (Fig. 1). It is unclear whether the blue light induction of Ca^2+^ release is acting via inhibition of ER Ca^2+^ refilling, such as by inhibiting an ER Ca^2+^ pump, or activation of an unknown ER localised Ca^2+^ channel.

**Fig. 1:**
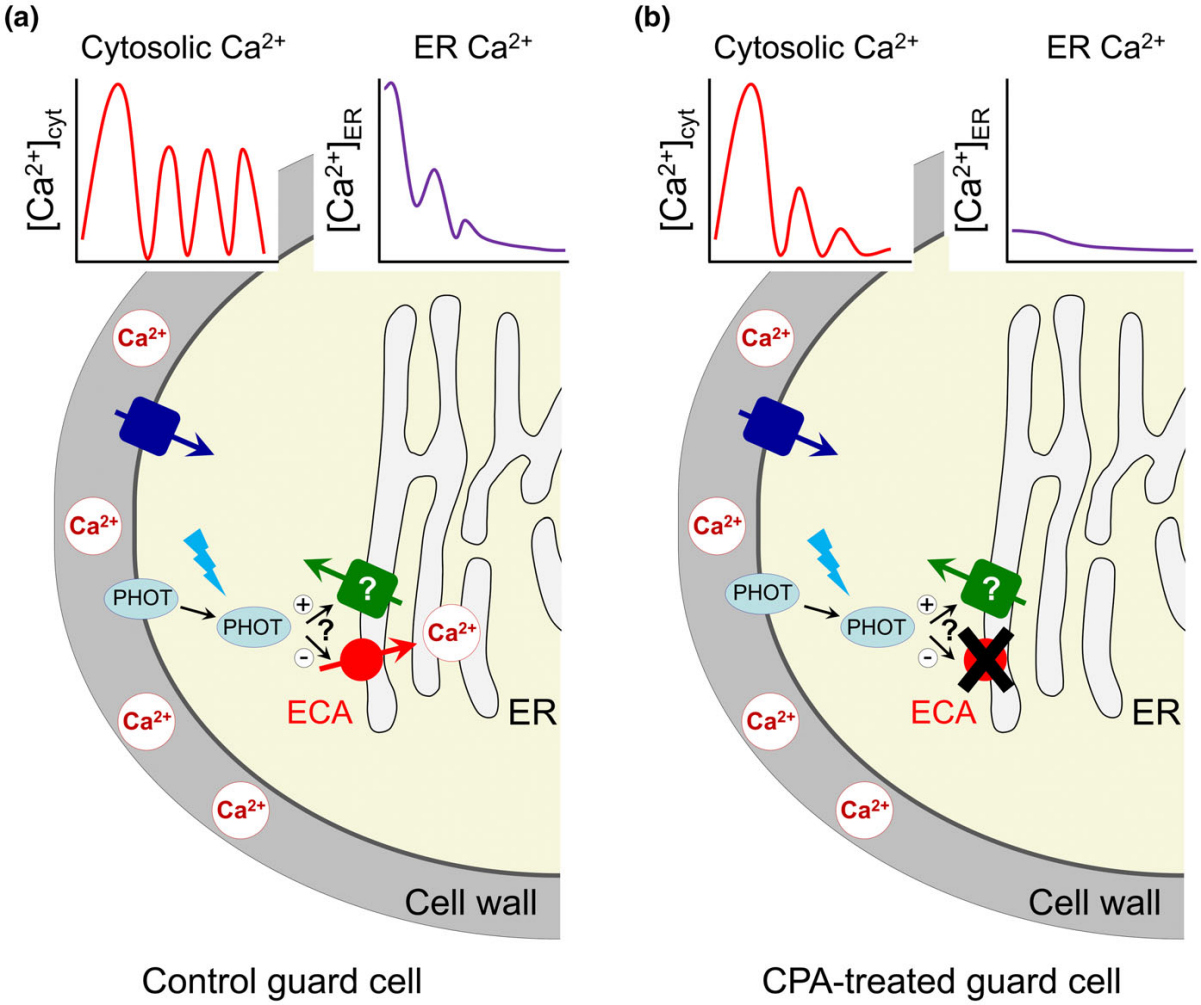
Model of spontaneous cytosolic Ca^2+^ ([Ca^2+^]_cyt_) oscillation generation in stomatal guard cells by interplay between apoplastic Ca^2+^ and endoplasmic reticulum (ER) Ca^2+^ ([Ca^2+^]_ER_). (a) In a control guard cell, apoplastic Ca^2+^ can enter the cytosol via plasma membrane localised Ca^2+^ permeable channels. This initiates a [Ca^2+^]_cyt_ oscillation, which is maintained over time by Ca^2+^ release from the ER, possibly via an unidentified Ca^2+^ permeable channel. This Ca^2+^ release will slowly deplete the ER Ca^2+^ pool. Under dark conditions the ER Ca^2+^ pool can be refilled by uptake via an ER‐type Ca^2+^‐ATPase (ECA). Blue light promotes ER Ca^2+^ release partly through the action of a phototropin (PHOT), which may positively regulate the ER Ca^2+^ release channel and/or negatively regulate the ECA. (b) Following cyclopiazonic acid treatment, ECA activity is inhibited and the ER Ca^2+^ pool cannot be filled, preventing the [Ca^2+^]_cyt_ oscillation from being maintained over time.

Pathways for the transport of Ca^2+^ into the ER are well characterised and involve two classes of Ca^2+^ pump, the autoinhibited Ca^2+^‐ATPase (ACA) and the ER‐type Ca^2+^‐ATPase (ECA), which both have isoforms present at the ER membrane in Arabidopsis (Brownlee & Wheeler, 2025). Cyclopiazonic acid (CPA) is a specific inhibitor of ECAs and a widely used drug in the investigation of ER Ca^2+^ homeostasis. Grenzi *et al*. found that CPA treatment abolished the dark induced ER Ca^2+^ refilling, indicating that this occurred via ECA pumps, while treatment with the Ca^2+^ chelator EGTA showed that the apoplastic Ca^2+^ pool was a key source of Ca^2+^ for ER refilling. A further confirmation of the importance of the ER Ca^2+^ pool was the demonstration that CPA exposure substantially inhibited the maintenance of the spontaneous [Ca^2+^]_cyt_ oscillations (Fig. 1) but also induced stomatal opening in the dark. Although the CPA inhibitor experiments provide convincing evidence of the involvement of ECA pumps, future confirmation is needed, such as from mutant analysis of specific ER localised ECA isoforms and validation of ECA isoform expression in guard cells. Recent examination of an Arabidopsis mutant line (*aca1*/*aca2*/*aca7*) lacking all three ER localised ACA isoforms demonstrated that ER Ca^2+^ pump activity is a key component in modulating guard cell [Ca^2+^]_cyt_ oscillations, and ultimately that manipulation of ER Ca^2+^ homeostasis is a means to modify stomatal conductance and the water‐use efficiency of the plant (Jezek *et al*., 2021). As ECA pumps were presumably expressed in the *aca1*/*aca2*/*aca7* mutant line, this suggests that they are not functionally interchangeable with the ACA pumps; therefore, it will be interesting to determine whether ECA mutant plants possess distinct guard cell Ca^2+^ phenotypes.

The work by Grenzi *et al*. follows on from other recent studies that have further demonstrated the importance of the ER in plant cells as a source of Ca^2+^ for the generation of [Ca^2+^]_cyt_ elevations (Resentini *et al*., 2021a; Huang *et al*., 2024). The ER is a key component in the formation of Ca^2+^ oscillations in animal cells, with tightly coupled regulation between this intracellular Ca^2+^ source and the extracellular Ca^2+^ pool, and well characterised ER Ca^2+^ release pathways, including inositol triphosphate (IP_3_) induced Ca^2+^ release (Xiong *et al*., 2025). While there are many differences in the evolution and composition of a Ca^2+^ signalling toolkit between plants and animals (Edel *et al*., 2017; Brownlee & Wheeler, 2025), the importance of ER homeostasis is conserved but with some key differences in molecular components. For example, both IP_3_ receptors and ryanodine receptors are absent in higher plants. A major deficiency in our understanding of plant ER Ca^2+^ homeostasis therefore is the lack of molecular identity of a Ca^2+^ release channel. Further investigation of blue light induced ER Ca^2+^ release is a promising route to uncover the identity of an ER Ca^2+^ permeable channel protein.

This study exemplifies the benefits of simultaneous subcellular Ca^2+^ imaging in multiple locations to further enhance our understanding of Ca^2+^ oscillations and Ca^2+^ signalling mechanisms. The continued development of high sensitivity Ca^2+^ reporters targeted to different organelles and subcellular locations, coupled with the use of mutant lines of candidate genes, will allow many unanswered questions to be addressed, not just in model systems like the stomatal guard cells, but in other cell types and across different plant species. These include elucidating potential crosstalk in signalling and Ca^2+^ transfer between different subcellular compartments. There is also potential for the continued use of biosensor tools to correlate and compare the dynamics of Ca^2+^ with other signalling molecules, including reactive oxygen species, pH, hormones, and more, both within cells and across multiple cells at whole tissue and whole plant levels (Rowe *et al*., 2025).

## Disclaimer

The New Phytologist Foundation remains neutral with regard to jurisdictional claims in maps and in any institutional affiliations.
